# Cardiac Changes in Patients on Long-Term Parenteral Nutrition

**DOI:** 10.3390/nu11071587

**Published:** 2019-07-13

**Authors:** Lidia Santarpia, Roberta Esposito, Andrea Vaccaro, Lucia Alfonsi, Maria Carmen Pagano, Ciro Santoro, Maurizio Marra, Franco Contaldo, Maurizio Galderisi, Fabrizio Pasanisi

**Affiliations:** 1 Internal Medicine and Clinical Nutrition, Department of Clinical Medicine and Surgery, Federico II University, Via Pansini, 580131 Naples, Italy; 2 Cardiovascular Emergencies and Onco-hematologic Complications, Department of Advanced Biomedical Sciences, Federico II University Medical School, 80131 Naples, Italy

**Keywords:** short bowel syndrome, home parenteral nutrition, transthoracic echocardiography, cardiac changes

## Abstract

Patients with short bowel syndrome (SBS) on long-term home parenteral nutrition (HPN) chronically receive high fluid volumes directly into the right atrium (RA) through the superior vena cava. We retrospectively evaluated cardiac function measured by routine transthoracic echocardiography (TTE) in a population of 26 SBS patients on long-term HPN and compared their data on echocardiograph-derived right heart structure and function, with those of a control group of 26 patients also bearing a central venous catheter (CVC) for other reasons. Results showed that body weight and BMI were significantly higher in the control group. The echocardiographic estimate of RA pressure was higher in HPN patients than in controls (*p* = 0.01). An increased estimate of RA pressure indicates the need to consider TTE in the follow-up of long-term HPN patients to detect functional impairment early.

## 1. Introduction

Generally, in clinical practice, the right atrium (RA) and right ventricle (RV) are less often considered than the other two cardiac chambers, and their role in cardiac pathophysiology remains insufficiently explored [1,2]. Transthoracic echocardiography (TTE) is an easy, noninvasive technique for right heart evaluation, despite its complex shape and retrosternal position, which limits an accurate ultrasound approach to the right section [3,4,5].

Patients with short bowel syndrome (SBS) on long-term home parenteral nutrition (HPN) chronically receive considerable fluid volumes directly into the RA through the superior vena cava (SVC), generally overnight [6].

It is conceivable that, in the long term, this fluid overload could exert a significant influence on the structure and function of right chambers. To the best of our knowledge, the possible hemodynamic changes of the right heart in patients with SBS undergoing long-term HPN have not been investigated.

The present retrospective study was designed to analyze the right heart chamber phenotype of SBS patients on long-term HPN in comparison with a control population bearing a central venous catheter (CVC) for oncologic reasons.

## 2. Methods

### 2.1. Study Population

All adult patients with SBS on long-term home parenteral nutrition (HPN) for a median of 70 months (range 19–96) who underwent a TTE between 2010 and 2016 were considered for the study. 

Clinically stable and otherwise healthy patients with a recent diagnosis of a first-stage oncological disease, bearing a CVC and undergoing a TTE before starting their chemotherapy program, served as a control group.

SBS patients with any kind of pulmonary or heart disease, including symptomatic heart failure, coronary artery disease, primary cardiomyopathies, valvular heart diseases, atrial fibrillation, and inadequate cardiac imaging were excluded from the study.

### 2.2. TTE

All image acquisitions were performed by an experienced operator with a GE Vivid 7 ultrasound machine (GE Healthcare, Horten, Norway) equipped with a 3.5 MHz transducer according to the standards of our laboratory [7,8]. RV global systolic function was assessed by measuring M-mode-derived tricuspid annular plane systolic excursion (TAPSE, mm) in the apical four-chamber view. RV transverse basal, middle, and longitudinal diameters; RA transverse diameter; and RA volume were measured in an apical four-chamber view. RV diameters and RA measurements were indexed for body surface area (BSA). Continuous-wave (CW) Doppler of tricuspid regurgitation (TR) was used to determine the pressure gradient between the right ventricle and right atrium. RA pressure (RAPr) was estimated from the quantitative assessment of the size and the collapsibility index of inferior vena cava (CI_ivc) (3 or 8 or 15 mmHg in relation to size and inspiration at rest and during forced inhalation), and this value was added to the peak tricuspid regurgitation gradient to estimate systolic pulmonary arterial pressure (sPAP) according to ESC guidelines [9].

### 2.3. Statistical Analysis

Statistical analysis was performed using SPSS release 18 (SPSS Inc, Chicago, IL, USA) software. Data are presented as the mean ± standard deviation (SD). The comparison between continuous variables of the two groups was performed through ANOVA. Least squares linear regression was used to evaluate univariate correlates of a given variable. Multiple linear regression analyses were used to identify the independent contributors of variables in the pooled population. Collinearity was considered acceptable and regression model stable for variance inflation factor <3. Differences between groups were considered significant when *p* < 0.05.

## 3. Results

Twenty-six patients (11 M, 15 F, 49.65 ± 18.81 years, 53.05 ± 12.70 kg, body mass index (BMI) 19.71 ± 3.86 kg/m^2^) with SBS who were on long-term HPN were evaluated; 26 age- and sex-matched patients (11 M, 15 F, 50.54 ± 17.24 years, 62.50 ± 12.47 kg, BMI 22.83 ± 4.03 kg/m^2^) bearing a central venous catheter for chemotherapy were used as control subjects.

Table 1 describes the demographic and anthropometric characteristics of the two groups. As expected, body weight, BMI, and body surface area (BSA) were significantly higher in the control group. Diastolic blood pressure was lower in HPN patients (*p* = 0.02).

**Figure 1:**
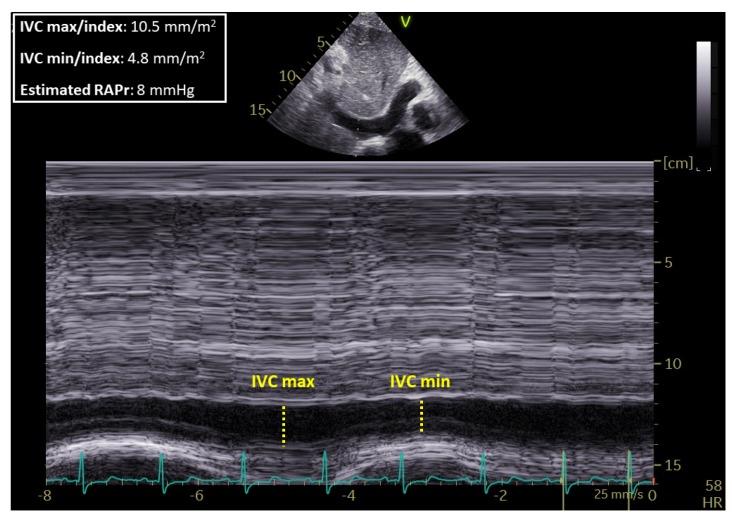
IVCmax: Inferior vena cava maximal diameter, IVCmin index: Inferior vena cava minimal diameter and estimated right atrium pressure (RAPr) in a patient with short bowel syndrome (SBS) on long-term home parenteral nutrition (HPN).

Table 2 shows the main parameters of the right heart evaluated by TTE in the two groups of patients. The estimated RA pressure (RAPr) (*p* = 0.01) and the maximal diameter of IVC (IVCmax) (*p* = 0.006) were higher in HPN patients than in controls, while the CI_ivc was lower than in controls (*p* = 0.015). Figure 1 depicts the IVC max index and the IVC min index and the estimated RAPr in a patient on HPN.

By performing separate multiple regression analyses in the pooled population (patients with parental nutrition + controls), after adjusting for age and BMI, the parenteral nutrition state remained independently associated with IVCmax index (standardized β coefficient = 0.427, *p* < 0.0001) and with RAPr (β = 0.35, *p* = 0.026). In addition, no correlation of cardiac parameters explored with daily parenteral volumes infused, days of infusion per week, and duration of parenteral nutrition emerged in the setting of patients undergoing parenteral nutrition.

## 4. Discussion

Home parenteral nutrition is a life-saving long-term therapy for patients with intestinal insufficiency and short bowel syndrome (SBS), who may need this treatment for the rest of their lives.

In our experience, some patients have been on long-term HPN for 20 years [10,11].

According to the ESPEN guidelines, HPN patients need approximately 35 mL fluids/kg body weight/day, but their requirements may widely vary depending on individual factors, including daily fluid losses and co-morbid conditions [12]. Moreover, in their clinical history, these patients may face dehydration and/or volume overload which can also rapidly occur [13,14].

Patients with moderate SBS require 1.5–2 L of intravenous fluid/day and are generally infused overnight to allow for their normal daylight activities. Some patients, particularly those with an end-jejunostomy, experience high stomal outputs (up to 7 L/day), which consequently increase their parenteral fluid needs [6,15].

Parenteral nutrition requires a careful balance of micro and macronutrient composition and volume and has to be prescribed by specialized physicians. Infused volumes may vary widely according to the different anatomical conditions and even in the same patient depending on the specific clinical setting; for these reasons, patients must be carefully monitored [16].

Currently, studies on possible cardiac overload have not been carried out in patients with SBS on HPN, but few studies are available on patients on hemodialysis—a population at high risk of pulmonary hypertension and mortality [17].

In the present study, for the first time, we investigated the possible impact of a chronic preload increase on RA and RV dimensions and function in patients on long-term HPN. Toward this aim, we used data from TTE, whose measurements—despite some levels of inaccuracy in comparison with the gold-standard technique, i.e., cardiac MRI [18,19,20]—provide meaningful clinical and prognostic information in the clinical setting.

The collected findings demonstrate that our SBS patients on long-term HPN had an increased noninvasive estimate of RAPr and a higher IVCmax than controls, while the CI_ivc was lower. At the present time, the possible mechanisms underlying the increased RAPr of these patients are not clear.

Conversely, sPAP was not significantly higher than that observed in the control group. Accordingly, we can theorize that increased RAPr is not associated to pulmonary hypertension in these patients and could be due to a chronic preload increase, due to fluid infusion. In fact, although the diameters of both right atrium and right ventricle did not appear to be significantly increased in comparison with the normal controls, the larger IVC diameter found can be considered as an initial marker of increased preload in this particular population.

Notably, the impact of parenteral nutrition state on IVC diameter and RAPr remained significant even after adjustment for age and BMI—both recognized determinants of right heart structure and function. In addition, received parenteral volumes, days of infusion per week, and duration of parenteral nutrition dependence did not influence the obtained results.

## 5. Conclusions

Patients with SBS on long-term HPN appear to be at high risk of hemodynamic changes and high probability of increased RAPr.

Presently, TTE evaluation is only used to explore cardiac valves in case of catheter-related bloodstream infection [12]. According to our findings in this study, a scheduled TTE could be of benefit to these patients in monitoring RAPr over time and in preventing possible long-term overload complications.

Due to the low number of patients studied so far, it is not possible to identify those at a higher risk, which could be candidates for specific exploratory studies. An extension of this pilot study combined with additional and multicenter studies would be useful to assess cardiac changes in patients undergoing long term HPN on a wider scale.

## Figures and Tables

**Table 1:** Characteristics of the two groups: 26 short bowel syndrome (SBS) patients on long-term home parenteral nutrition (HPN) and 26 patients (controls) bearing a central venous catheter (CVC) to start chemotherapy.

Variable	HPN Patients (*n* = 26)	Controls (*n* = 26)	*p*-Value
Sex (M/F)	11/15	11/15	
Age (years)	49.65 ± 18.81	50.44 ± 17.24	0.81
Body Weight (kg)	53.05 ± 12.70	62.50 ± 12.47	0.01
Height (cm)	163.57 ± 8.70	165.34 ± 9.16	0.99
BMI (kg/m^2^)	19.71 ± 3.86	22.83 ± 4.03	0.01
BSA (m^2^)	1.57 ± 0.23	1.68 ± 0.18	0.05
SBP (mmHg)	117.88 ± 30.38	125.63 ± 14.01	0.34
DBP (mmHg)	68.06 ± 12.76	77.08 ± 9.65	0.02
HR (bpm)	80.46 ± 19.35	80.73 ± 14.90	0.87
HPN duration (months)	70 (19–96)		
Iv volumes (mL)	1621 ± 501		
Iv volumes/Body Weight (mL/kg)	29.34 ± 12.35		

HPN patients: patients on home parenteral nutrition; ns: not significant; BMI: body mass index; BSA: body surface area; SBP: systolic blood pressure; DBP: diastolic blood pressure; HR: heart rate (reported as median and range); bpm (beats per minute); Iv volumes: volumes of intra-venous therapy; Iv volumes/Body Weight: intra-venous therapy volumes adjusted for body weight.

**Table 2:** Main echocardiographic parameters of the two groups: 26 SBS patients on long-term home parenteral nutrition (HPN) and 26 patients (controls) bearing a central venous catheter (CVC) to start chemotherapy.

Variable	HPN Patients (*n* = 26)	Controls (*n* = 26)	*p*-Value
TRGrad. (mmHg)	25.44 ± 9.86	22.62 ± 5.72	0.71
sPAP (mmHg)	32.70 ± 11.51	28.97 ± 6.60	0.51
IVCmax_Index (mm/m^2^)	10.3 ± 2.84	7.80 ± 2.25	0.006
IVCmin_Index (mm/m^2^)	4.46 ± 3.81	2.38 ± 2.61	0.04
CI_ivc (%)	58.18 ± 33.87	70.41 ± 29.46	0.27
RAPr (mmHg)	7.39 ± 2.96	5.57 ± 1.62	0.01
RA volume (mL)	28.29 ± 13.85	28.90 ± 10.89	0.99
RAvol_Index (mL/m^2^)	17.53 ± 6.22	17.00 ± 5.63	0.98
RAdiam. (mm)	3.28 ± 0.58	3.19 ± 0.62	0.84
RAdiam Index (mm/m^2^)	2.11 ± 0.38	1.93 ± 0.36	0.15
RVDbasal diam (mm)	32.99 ± 5.83	33.76 ± 5.26	0.94
RVDbasal diam Index (mm/m^2^)	21.13± 3.20	20.06 ± 2.77	0.62
RVDmean (mm)	29.42 ± 5.62	29.58 ± 6.59	1.00
RVDmean Index (mm/m^2^)	18.84 ± 3.13	17.47 ± 2.92	0.46
RVDlong (mm)	56.96 ± 6.58	63.62 ± 8.22	0.002
RVDlong Index (mm/m^2^)	36.58 ± 4.00	37.78 ± 3.80	0.78
TAPSE (mm)	21.46 ± 3.90	22.73 ± 3.46	0.42

TRGrad = Tricuspid regurgitation gradient; sPAP = Systolic pulmonary arterial pressure; IVCmax_Index = Inferior vena cava indexed maximal diameter; IVCmin_Index = Inferior vena cava indexed minimal diameter; CI_ivc (%): inferior vena cava collapsibility index; RAPr = right atrium pressure; RA volume = right atrium volume; RAvol_Index = right atrium indexed volume¸ RAdiam = right atrium diameter; RAdiam_Index = right atrium indexed diameter; RVDbasal diam = right ventricle basal diameter, RVDbasal diam Index = right ventricle basal indexed diameter; RVDmean (mm) = right ventriclemean diameter; RVDmean Index (mm)) = right ventricle indexed mean diameter; RVDlong = right ventricle longitudinal diameter; RVDlong_Index = right ventricle longitudinal indexed diameter; TAPSE: tricuspid annular plane systolic excursion.

## References

[1] Goor D.A., Lillehei C.W. (1975). Congenital malformations of the heart. Congenital Malformations of the Heart: Embryology, Anatomy, and Operative Considerations.

[2] Ho S.Y., Nihoyannopoulos P. (2006). Anatomy, echocardiography, and normal right ventricular dimensions. Heart.

[3] Voelkel N.F., Quaife R.A., Leinwand L.A., Barst R.J., McGoon M.D., Meldrum D.R., Dupuis J., Long C.S., Rubin L.J., Smart F.W. (2006). Right ventricular function and failure: Report of a National Heart, Lung, and Blood Institute Working Group on Cellular and Molecular Mechanisms of Right Heart Failure. Circulation.

[4] Lorenz C.H., Walker E.S., Morgan V.L., Klein S.S., Graham T.P. (1999). Normal human right and left ventricular mass, systolic function, and gender differences by cine magnetic resonance imaging. J. Cardiovasc. Magn. Reson..

[5] Chin K.M., Kim N.H., Rubin L.J. (2005). The right ventricle in pulmonary hypertension. Coron. Artery Dis..

[6] Matarese L.E. (2013). Nutritional and fluid optimization for patients with Short Bowel Syndrome. JPEN J. Parenter. Enter. Nutr..

[7] Lembo M., Esposito R., Santoro C., Lo Iudice F., Schiano-Lomoriello V., Fazio V., Grimaldi M.G., Trimarco B., de Simone G., Galderisi M. (2018). Three-dimensional echocardiographic ventricular mass/end-diastolic volume ratio in native hypertensive patients: Relation between stroke volume and geometry. J. Hypertens..

[8] Buonauro A., Galderisi M., Santoro C., Canora A., Bocchino M.L., Lo Iudice F., Lembo M., Esposito R., Castaldo S., Trimarco B. (2017). Obstructive sleep apnoea and right ventricular function: A combined assessment by speckle tracking and three-dimensional echocardiography. Int. J. Cardiol..

[9] Galiè N., Humbert M., Vachiery J.L., Gibbs S., Lang I., Torbicki A., Simonneau G., Peacock A., VonkNoordegraaf A., Beghetti M. (2016). ESC Scientific Document Group. 2015 ESC/ERS Guidelines for the diagnosis and treatment of pulmonary hypertension: The Joint Task Force for the Diagnosis and Treatment of Pulmonary Hypertension of the European Society of Cardiology (ESC) and the European Respiratory Society (ERS): Endorsed by: Association for European Paediatric and Congenital Cardiology (AEPC), International Society for Heart and Lung Transplantation (ISHLT). Eur. Heart J..

[10] Santarpia L., Pagano M.C., Pasanisi F., Contaldo F. (2014). Home artificial nutrition: An update seven years after the regional regulation. Clin. Nutr..

[11] Violante G., Alfonsi L., Santarpia L., Cillis M.C., Negro G., De Caprio C., Russo N., Contaldo F., Pasanisi F. (2006). Adult home parenteral nutrition: A clinical evaluation after a 3-year experience in a Southern European centre. Eur. J. Clin. Nutr..

[12] Pironi L., Arends J., Bozzetti F., Cuerda C., Gillanders L., Jeppesen P.B., Joly F., Kelly D., Lal S., Staun M. (2016). ESPEN guidelines on chronic intestinal failure in adults. Clin. Nutr..

[13] Bielawska B., Allard J.P. (2017). Parenteral Nutrition and Intestinal Failure. Nutrients.

[14] Wanten G., Calder P.C., Forbes A. (2011). Managing adult patients who need home parenteral nutrition. BMJ.

[15] O’Keefe S.J., Peterson M.E., Fleming C.R. (1994). Octreotide as an adjunct to home parenteral nutrition in the management of permanent end-jejunostomy syndrome. JPEN J. Parenter. Enter. Nutr..

[16] Tappenden K.A. (2014). Intestinal adaptation following resection. JPEN J. Parenter. Enter. Nutr..

[17] Reque J., Quiroga B., Ruiz C., Villaverde M.T., Vega A., Abad S., Panizo N., López-Gómez J.M. (2016). Pulmonary hypertension is an independent predictor of cardiovascular events and mortality in haemodialysis patients. Nephrology.

[18] Dini F.L., Galderisi M., Mondillo S., De Tommasi S.M. (2004). The right ventricle: Role of Doppler echocardiography in the clinical practice. Ital. Heart J. Suppl..

[19] Whitlock M., Garg A., Gelow J., Jacobson T., Broberg C. (2010). Comparison of left and right atrial volume by echocardiography versus cardiac magnetic resonance imaging using the area-length method. Am. J. Cardiol..

[20] Lai W.W., Gauvreau K., Rivera E.S., Saleeb S., Powell A.J., Geva T. (2008). Accuracy of guideline recommendations for two-dimensional quantification of the right ventricle by echocardiography. Int. J. Cardiovasc. Imaging.

